# Association between visceral adiposity index and osteoarthritis in U.S. adults aged 50 and older: a cross-sectional study

**DOI:** 10.3389/fnut.2025.1542937

**Published:** 2025-05-13

**Authors:** Zitian Wang, Guang Peng, Yuquan Jiang, Jintao Qu, Fengfu Wu

**Affiliations:** Department of Orthopedics, Burn and Plastic Surgery, The 925th Hospital, Guiyang, China

**Keywords:** visceral adiposity index, osteoarthritis, NHANES, association, cross-sectional analysis

## Abstract

**Background:**

Existing evidence linking visceral adiposity index (VAI) to osteoarthritis (OA) remains limited and requires further investigation. This study aimed to evaluate the potential relationship between higher VAI scores and an increased risk of OA.

**Methods:**

A retrospective cross-sectional analysis was conducted using data from 9,464 participants aged 50 and older, sourced from the 2011 to 2018 National Health and Nutrition Examination Survey (NHANES). The VAI was categorized into three tertiles, with the first tertile (T1) representing the lowest VAI and third tertile (T3) the highest. Weighted logistic regression was employed to examine the association between VAI and OA. To explore potential non-linear relationships, smoothed curve fitting and threshold effect analyses were performed. Subgroup analyses were performed to validate these findings.

**Results:**

The average age of the study population was 63.16 ± 9.05 years, and 47.22% were male. After adjusting for confounding factors, a statistically significant positive correlation was observed between VAI and OA risk (OR = 1.03, 95% CI: 1.01–1.06, *P* < 0.01). Participants in the highest VAI tertile exhibited a 35% greater likelihood of developing OA compared to those in the lowest tertile (OR = 1.35, 95% CI: 1.06–1.70, *P* = 0.015). Furthermore, multivariate restricted cubic spline (RCS) regression analysis revealed a non-linear relationship (non-linear *P* < 0.05) with a threshold effect at a VAI value of 3.9. Subgroup analyses showed no significant interaction effects (all *P*-values for interaction > 0.05).

**Conclusion:**

This study highlights a significant association between elevated VAI and an increased risk of developing OA in individuals aged 50 and older. These results emphasize the potential of the VAI as a risk factor for OA and warrant further research to explore its role in prevention and management strategies in older populations.

## 1 Introduction

Osteoarthritis (OA) is a widespread chronic joint condition that primarily affects middle-and older adults worldwide (1, 2). Approximately 250 million people worldwide are affected by OA, with 18% of women and 9.6% of men over 60 years of age experiencing symptomatic OA (3). Data from a recent National Health Interview Survey in the United States suggest that approximately 14 million people suffer from symptomatic knee OA, of which approximately 3 million are from minority populations (4), posing a considerable challenge to public health (5). OA is characterized by several pathological changes, including gradual cartilage degeneration, synovial inflammation, osteophyte formation, and alterations in subchondral bone structure (6, 7) Moreover, OA also involves tissue changes in the menisci, tendons, and ligaments, as well as infrapatellar fat pad, which contribute to local inflammation associated with increased pain, functional impairment, and inflammatory markers release (8). While the precise origins of OA remain unclear, it is understood that several factors are linked to its onset and progression. These factors include age, female sex, genetic predisposition, mechanical stress, metabolic imbalances, prior joint injury (9). Furthermore, growing evidence suggests that obesity plays a significant role in the pathogenesis of OA. Specifically, obesity triggers a low-grade systemic inflammatory response, characterized by the production and release of various adipocytokines, which may further contribute to the development of OA (10). Consequently, early detection and management of OA are crucial to reduce its prevalence and improve patient outcomes.

Visceral Adiposity Index (VAI) is a comprehensive metric that combines body mass index (BMI), waist circumference (WC), triglyceride (TG), and HDL cholesterol (HDL-C) to assess visceral fat (11). Compared to traditional measures such as BMI, waist circumference, and waist-to-height ratio, the VAI has demonstrated superior ability in evaluating visceral fat distribution and dysfunction in adults (12, 13). Clinical studies have shown that the VAI is effective in identifying individuals at increased risk for metabolic disorders associated with visceral obesity, such as insulin resistance, dyslipidemia, and cardiovascular risk factors (14–16). Visceral fat accumulation increases with age, particularly in older adults (17). Thus, VAI is a promising tool for predicting the development of OA.

However, the relationship between VAI and OA in middle-aged and older adults remains unclear. To address this gap, we conducted an investigation into the association between VAI and OA among U.S. adults aged 50 and older. Specifically, we aimed to (1) assess the correlation between the VAI and OA, (2) examine non-linear associations, and (3) explore subgroup differences.

## 2 Materials and methods

### 2.1 Study population

The NHANES, which is overseen by the Centers for Disease Control and Prevention, is a large-scale study designed to evaluate the health and nutritional status of the U.S. population. The assessment was conducted using a combination of interviews, physical examinations, and laboratory tests. The details of the study methodology, including data collection and sample weighting, can be found at http://www.cdc.gov/nchs/nhanes.html. The NHANES protocol has been approved by the Research Ethics Review Committee of the National Center for Health Statistics, and written informed consent was obtained from all participants. Our analysis, based on secondary NHANES data, was exempt from institutional review (18).

**FIGURE 1:**
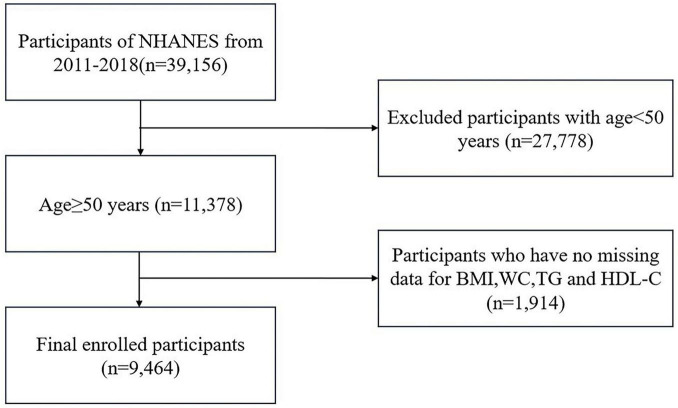
Flowchart for inclusion and exclusion criteria.

In this investigation, 39,156 individuals participated in four NHANES cycles from 2011 to 2018. Individuals under the age of 50 years (*n* = 27,778) and those with missing VAI and OA data (total, *n* = 1, 914; BMI = 719; WC = 655; TG = 536; HDL-C = 4; OA = 0) were excluded. In total, 9,464 participants aged 50 and older were included in the final analysis (Figure 1).

### 2.2 Outcome and exposure factor

The primary outcome of this study was OA diagnosis. To assess OA, individuals aged 20 years and older were asked two specific questions about their arthritis status (19). The first question was, “Has a doctor or other healthcare professional ever told you that you have arthritis?” Those who answered “yes” were then asked, “What type of arthritis was it?” Participants identifying their condition as “Osteoarthritis or degenerative arthritis” were categorized as having OA. The self-reported “definite” OA aligns with clinical diagnoses in up to 81% of cases, indicating a high level of accuracy in self-reported diagnoses (20).

In this analysis, the VAI served as the main exposure variable. VAI was calculated using sex-specific formulas according to previously reported equations (21): for men, the equation was waist circumference/[39.68 + (1.88 × BMI)] × (triglycerides/1.03) × (1.31/HDL-C); for women, the formula was waist circumference/[36.58 + (1.89 × BMI)] × (triglycerides/0.81) × (1.52/HDL-C), where both TG and HDL levels are expressed in mmol/L (22). The participants were divided into tertiles based on their VAI values for further statistical analyses.

### 2.3 Selection of covariates

During the home interviews, the NHANES staff gathered data through questionnaires that covered various participant characteristics, including age, sex, race/ethnicity, education, marital status, family income, smoking habits, alcohol consumption, sleep disorders, and the presence of diabetes. Race/ethnicity was categorized as non-Hispanic White, non-Hispanic Black, Mexican American, and other ethnic groups. Education levels were grouped as less than high school, high school or equivalent, and greater than high school. Family income was classified based on the poverty income ratio (PIR) into low (< 1.30), middle (1.30y income was clas (≥ 3.50) income brackets. Alcohol intake was categorized by whether participants consumed ≥ 4 drinks per day, whereas smoking status was categorized as former, current, or never.

As part of the laboratory examinations during the 2011–2018 NHANES cycles, blood samples were collected following stringent protocols for analysis. At baseline, the following biomarkers were measured: alanine aminotransferase (ALT), aspartate aminotransferase (AST), alkaline phosphatase (ALP), blood urea nitrogen (BUN), total calcium, total cholesterol (TC), serum glucose, and serum uric acid (SUA).

### 2.4 Statistical analyses

To analyze the NHANES dataset, incorporation of sampling weights and design variables is essential because failure to do so may result in biased estimates and inflated significance levels. Therefore, our analysis followed NHANES guidelines by employing a complex sampling design and applying the appropriate sampling weights. The data used in this study were obtained from home interviews and a Mobile Examination Center (MEC) during the NHANES survey. In accordance with the NHANES guidelines on survey sample weights, the MEC weights were used in this analysis, with 2011–2018 weights calculated as 1/4 of the 2-year MEC weight (23).

Continuous variables with a normal distribution were expressed as the mean ± standard deviation (SD), whereas categorical variables were presented as frequencies and percentages. Categorical variables were analyzed using the chi-square test and normally distributed continuous variables were analyzed using one-way ANOVA of variance to compare the differences between the different VAI groups.

To assess the effect of VAI on OA, binary logistic regression models were applied to calculate odds ratios (OR) and 95% confidence intervals (CI), while adjusting for relevant covariates. The VAI was treated as both a continuous and a categorical variable with three levels. Covariate selection has been described in the existing literature (24). Three regression models were used: Model 1 was unadjusted; Model 2 was adjusted for age, sex, race, education, marital status, PIR, smoking status, alcohol intake, moderate recreational activity, sleep disturbances, and diabetes; and Model 3 was additionally adjusted for ALT, AST, ALP, BUN, total calcium, TC, glucose, and SUA levels.

We employed a restricted cubic spline model to explore the potential non-linear dose-response relationships between VAI and OA, assessing non-linearity by including a quadratic term in the regression. Based on the results of the smoothing curve, a two-piecewise linear regression model was used to investigate the threshold effects after adjusting for confounding variables. Predefined subgroup analyses were performed based on the clinical interest and previous scientific literature.

We employed statistical imputation methods to address missing data for covariates. For continuous variables, missing values were imputed using either the mean or the median, enabling us to retain incomplete data in our analysis. Additionally, multiple imputation (MI) with five replications and the chained equations method, implemented through the R “mice” package, was applied as part of sensitivity analyses to further account for missing data.

All statistical analyses were conducted using R Statistical Software (Version 4.2.2; The R Foundation)^1^ and the Free Statistics Analysis Platform (Version 2.0; Beijing, China).^2^ The free statistical analysis platform provides an intuitive interface for common statistical analyses and data visualization using R as the core statistical engine and Python as the graphical user interface. This platform facilitates reproducible analyses and interactive data exploration. Differences were considered statistically significant at a two-sided *p*-value < 0.05.

## Results

A total of 9,464 participants were enrolled in this study following a thorough screening process based on predefined inclusion and exclusion criteria. Participants were divided into tertiles based on their VAI scores. The average age of the cohort was 63.16 ± 9.05 years, with 47.22% identifying as male. As the VAI values increased, a concurrent increase was observed in the prevalence of diabetes; incidence of sleep disturbances; and levels of ALT, ALP, TC, blood glucose, and SUA. In contrast, the proportion of individuals with an education beyond high school declined with increasing VAI. Furthermore, participants in the highest VAI tertile (T3) were more likely to report smoking and alcohol misuse and had lower rates of engagement in moderate recreational activities than those in the lower tertiles (T1 and T2). A detailed summary of the baseline characteristics of the VAI tertiles is presented in Table 1.

**TABLE 1:** Weighted characteristics of the study population based on visceral adiposity index tertiles.

Visceral adiposity index	Total	T1 (< 1.32)	T2 (1.32–2.59)	T3 (> 2.59)	*p*-value
Number of subjects (*n*)	9,464	3,155	3,154	3,155	
Age	63.16 ± 9.05	63.31 ± 9.12	63.48 ± 9.05	62.69 ± 8.95	0.034
Gender (%)					<0.001
Male	4,675 (47.22)	1,752 (51.25)	1,464 (42.91)	1,459 (47.51)	
Female	4,789 (52.78)	1,403 (48.75)	1,690 (57.09)	1,696 (52.49)	
Race (%)					<0.001
Non-Hispanic White	3,806 (73.48)	1,179 (72.77)	1,285 (73.56)	1,342 (74.12)	
Non-Hispanic Black	2,124 (9.34)	1,054 (13.69)	647 (8.54)	423 (5.73)	
Mexican American	1,172 (5.16)	242 (3.29)	404 (5.35)	526 (6.87)	
Other race/ethnicity	2,362 (12.02)	680 (10.24)	818 (12.54)	864 (13.28)	
Education level (%)					<0.001
Less than high school	2,408 (14.48)	679 (11.16)	822 (15.52)	907 (16.77)	
High school	2,195 (23.54)	727 (21.86)	720 (22.91)	748 (25.87)	
More than high school	4,861 (61.99)	1,749 (66.98)	1,612 (61.57)	1,500 (57.35)	
Marital status (%)					0.686
With partner	5,648 (65.90)	1,846 (66.74)	1,888 (65.55)	1,914 (65.41)	
Single	3,816 (34.10)	1,309 (33.26)	1,266 (34.45)	1,241 (34.59)	
PIR (%)					<0.001
<1.3	2,523 (16.10)	709 (12.10)	850 (16.54)	964 (19.70)	
1.3–3.5	4,261 (40.20)	1,407 (38.68)	1,453 (41.02)	1,401 (40.90)	
>3.5	2,680 (43.70)	1,039 (49.22)	851 (42.45)	790 (39.40)	
Smoker (%)					0.004
Current	1,566 (15.45)	527 (14.40)	482 (14.28)	557 (17.72)	
Ever	3,020 (33.13)	1,000 (32.69)	995 (31.63)	1,025 (35.11)	
Never	4,878 (51.41)	1,628 (52.91)	1,677 (54.09)	1,573 (47.17)	
Drinker (%)					0.048
Yes	1,447 (15.22)	484 (13.56)	479 (14.81)	484 (17.32)	
No	8,017 (84.78)	2,671 (86.44)	2,675 (85.19)	2,671 (82.68)	
Moderate recreational activities (%)					<0.001
Yes	3,645 (44.77)	1,398 (51.62)	1,199 (44.84)	1,048 (37.75)	
No	5,819 (55.23)	1,757 (48.38)	1,955 (55.16)	2,107 (62.25)	
Trouble sleeping (%)					0.001
Yes	3,016 (35.49)	889 (31.97)	1,017 (36.00)	1,110 (38.54)	
No	6,448 (64.51)	2,266 (68.03)	2,137 (64.00)	2,045 (61.46)	
Diabetes (%)					<0.001
Yes	2,122 (17.60)	454 (9.06)	707 (16.47)	961 (27.41)	
No	7,342 (82.40)	2,701 (90.94)	2,447 (83.53)	2194 (72.59)	
ALT (U/L)	23.85 ± 19.46	22.52 ± 14.61	23.26 ± 15.43	25.80 ± 26.13	<0.001
AST (U/L)	25.13 ± 14.63	25.34 ± 13.15	24.46 ± 12.47	25.60 ± 17.75	0.119
ALP (IU/L)	72.37 ± 25.07	68.81 ± 22.73	73.11 ± 27.49	75.22 ± 24.35	<0.001
Blood urea nitrogen (mmol/L)	5.66 ± 2.13	5.62 ± 1.99	5.59 ± 2.14	5.77 ± 2.23	0.08
Serum total calcium (mmol/L)	2.35 ± 0.09	2.34 ± 0.09	2.35 ± 0.09	2.35 ± 0.09	0.015
Cholesterol (mmol/L)	5.11 ± 1.13	5.01 ± 1.04	5.09 ± 1.12	5.23 ± 1.21	<0.001
Serum glucose (mmol/l)	5.95 ± 2.18	5.46 ± 1.36	5.77 ± 1.81	6.63 ± 2.92	<0.001
Serum uric acid (mmol/L)	327.27 ± 82.8	309.72 ± 79.1	324.37 ± 80.6	347.99 ± 84.2	<0.001

Mean ± SD for continuous variables: the *p*-value was calculated by the weighted linear regression model. (%) for categorical variables: the *p*-value was calculated by the weighted chi-square test. PIR, poverty income ratio; ALT, alanine aminotransferase; AST, aspartate aminotransferase; ALP, alkaline phosphatase.

In multivariable logistic regression analysis (Model 3 in Table 2), VAI, treated as a continuous variable, was positively associated with the likelihood of developing OA (OR = 1.03, 95% CI: 1.01–1.06, *P* = 0.007). Additionally, univariate logistic regression analysis revealed a significant positive association between VAI (categorized into tertiles) and OA risk. Specifically, comparing the highest tertile (T3) to the lowest (T1) yielded an odds ratio of 1.34 (95% CI: 1.09–1.64, *P* = 0.006). This relationship remained statistically significant after adjusting for confounders, with the adjusted odds ratio being 1.35 (95% CI: 1.06–1.70, *P* = 0.015), as shown in Model 3 of Table 2.

**TABLE 2:** Association between visceral adiposity index and prevalence of osteoarthritis.

	Model 1 (unadjusted)		Model 2		Model 3	
	OR (95% CI)	*p*-value	OR (95% CI)	*p*-value	OR (95% CI)	*p*-value
VAI	1.03 (1.00∼1.05)	0.025	1.03 (1.00∼1.05)	0.019	1.03 (1.01∼1.06)	0.007
**VAI categories**						
T1 (<1.31)	1 (Ref)		1 (Ref)		1 (Ref)	
T2 (1.31–2.56)	1.35 (1.16∼1.59)	<0.001	1.29 (1.09∼1.54)	0.004	1.31 (1.10∼1.56)	0.003
T3 (>2.56)	1.34 (1.09∼1.64)	0.006	1.30 (1.03∼1.63)	0.028	1.35 (1.06∼1.70)	0.015
*P* for trend		0.007		0.03		0.015

Model 1: no covariates were adjusted. Model 2: age, gender, race, education level, marital status, PIR, smoking status, alcohol abuse, moderate recreational activities, trouble sleeping, diabetes status were adjusted. Model 3: Model 2 + ALT, AST, ALP, blood urea nitrogen, serum total calcium, cholesterol, serum glucose, serum uric acid were adjusted. PIR, poverty income ratio; ALT, alanine aminotransferase; AST, aspartate aminotransferase; ALP, alkaline phosphatase.

**FIGURE 2:**
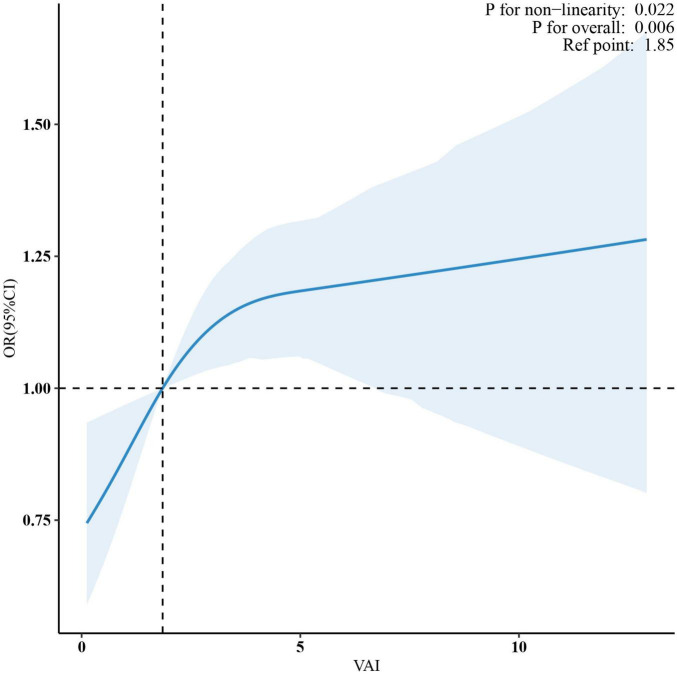
A non-linear pattern of the association between VAI and osteoarthritis (*p*-value for log-likelihood ratio test = 0.022) in a generalized additive model. The vertical dashed line marks the median point for VAI = 1.85, and non-linear relationships were detected with a breakpoint of 3.9. The model was adjusted for age, gender, race, education level, marital status, PIR, smoking status, alcohol abuse, moderate recreational activities, trouble sleeping, diabetes status, ALT, AST, ALP, blood urea nitrogen, serum total calcium, cholesterol, serum glucose and serum uric acid. VAI, visceral adiposity index; PIR, poverty income ratio; ALT, alanine aminotransferase; AST, aspartate aminotransferase; ALP, alkaline phosphatase.

Multivariate spline analysis revealed a non-linear relationship between VAI and OA (*P* for non-linearity = 0.022; Figure 2). Specifically, a significant positive association was observed up to a VAI of 3.9, beyond which the dose-response curve plateaued, indicating a lack of further significant correlation between VAI and OA (*P* = 0.162) (Table 3).

**TABLE 3:** Threshold effect analysis of visceral adiposity index and prevalence of osteoarthritis.

Risk of osteoarthritis	Adjusted OR (95% CI)	*p*-value
Fitting by the standard linear model	1.03 (1.01∼1.06)	0.007
**Fitting by the two-piecewise linear model**		
Inflection point	3.9	
Visceral adiposity index < 3.9	1.13 (1.02∼1.24)	0.022
Visceral adiposity index > 3.9	1.02 (0.99∼1.06)	0.162
*p* for log-likelihood ratio test		0.022

Age, gender, race, education level, marital status, PIR, smoking status, alcohol abuse, moderate recreational activities, trouble sleeping, diabetes status, ALT, AST, ALP, blood urea nitrogen, serum total calcium, cholesterol, serum glucose, serum uric acid were adjusted. PIR, poverty income ratio; ALT, alanine aminotransferase; AST, aspartate aminotransferase; ALP, alkaline phosphatase.

**FIGURE 3:**
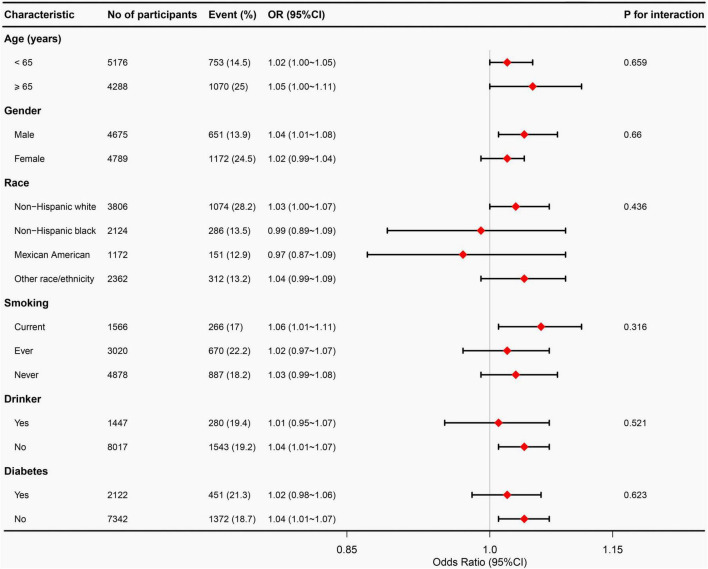
Subgroup analyses between VAI and osteoarthritis. Adjust for age, gender, race, education level, marital status, PIR, smoking status, alcohol abuse, moderate recreational activities, trouble sleeping, diabetes status, ALT, AST, ALP, blood urea nitrogen, serum total calcium, cholesterol, serum glucose, and serum uric acid. VAI, visceral adiposity index; PIR, poverty income ratio; ALT, alanine aminotransferase; AST, aspartate aminotransferase; ALP, alkaline phosphatase.

To examine potential effect modifiers on the relationship between VAI and OA, we conducted stratified analyses based on age, sex, race, smoking status, alcohol consumption, and diabetes status. However, no significant interactions were found in any of these subgroups, as all interaction *P*-values exceeded 0.05 in Figure 3.

*Post-hoc* sensitivity analyses using multiple imputations to address missing values for PIR (10.31%), total calcium (0.12%), AST (0.10%), ALT (0.10%), and ALP (0.10%) produced results that were consistent with those from the fully adjusted model (Table 4).

**TABLE 4:** ORs of five multiple imputation data.

	MI 1	MI 2	MI 3	MI 4	MI 5
VAI	1.03 (1.01∼1.06)	1.03 (1.01∼1.06)	1.03 (1.01∼1.06)	1.03 (1.01∼1.06)	1.03 (1.01∼1.06)
**VAI categories**					
T1	1 (Ref)	1 (Ref)	1 (Ref)	1 (Ref)	1 (Ref)
T2	1.31 (1.10∼1.56)	1.31 (1.10∼1.56)	1.32 (1.11∼1.56)	1.31 (1.10∼1.56)	1.31 (1.10∼1.56)
T3	1.34 (1.06∼1.70)	1.35 (1.07∼1.71)	1.35 (1.07∼1.71)	1.34 (1.06∼1.70)	1.34 (1.06∼1.70)

VAI, visceral adipose index; OR, odds ratio; MI, multiple imputation.

## Discussion

In this comprehensive cross-sectional analysis of U.S. adults aged 50 years and older, utilizing the NHANES 2011–2018 dataset, we identified a significant independent association between VAI and the risk of developing OA. Our findings revealed a dose-response relationship, indicating a non-linear connection between the VAI and OA risk (*P* for non-linearity < 0.05), with the association diminishing once the VAI values surpassed 3.9. Subgroup analyses further supported this association, reinforcing the relationship between higher VAI and increased OA risk across various demographic groups. This study is novel in that it uses the VAI as a predictor of OA, offering a new hypothesis for early OA screening and potential therapeutic interventions.

Although research on the relationship between the VAI and OA remains limited, BMI is frequently used to assess the risks of obesity and OA (25). A 22-year longitudinal study by Toivanen et al. demonstrated a significant association between a higher BMI and an increased risk of knee OA. Individuals with a BMI between 25.0 and 29.9 kg/m^2^ had a 70% higher risk of knee OA, and those with a BMI ≥ 30.0 kg/m^2^ had a 600% higher risk compared to those with a BMI < 25 kg/m^2^ (26). Similarly, Grotle et al. observed in a 10-year cohort study of 1,675 Norwegian adults that obese individuals (BMI ≥ 30) were 2.77 times more likely to develop knee OA than those with normal weight (27). Jiang et al. performed a systematic review and meta-analysis of 21 studies and concluded that a higher BMI was significantly associated with knee OA risk, with a 5-unit increase in BMI linked to a 35% greater risk (28). However, considerable heterogeneity was observed among the included studies.

As research has evolved, many scholars have questioned the adequacy of BMI for assessing OA risk, as BMI measures only total weight relative to height, without distinguishing between fat mass and lean body mass. It also does not account for the fat distribution, particularly abdominal fat (29). Alternative metrics should be considered to assess abdominal obesity more accurately. The VAI, which is derived from waist circumference, weight, height, and blood triglyceride and HDL cholesterol levels, offers significant advantages over BMI by reflecting the visceral fat content and its potential impact on bone metabolism-related diseases (30). Previous studies have linked VAI to several health conditions including diabetes, hyperuricemia, metabolic syndrome, hypertension, atherosclerosis, and heart failure (31, 32). While the relationship between VAI and OA is not well established, our study identified a significant association between elevated VAI and an increased risk of OA.

Furthermore, our study found a non-linear relationship and a threshold effect between VAI and OA, which aligns with the findings of other observational studies. For instance, Huang et al. identified a non-linear association between lipid accumulation products and OA risk, with a notable threshold effect of 120.00 cm × mmol/L (33). Similarly, a recent study analyzing the triglyceride-glucose index found significant associations and threshold effects in arthritis (34). In contrast, Wang et al. reported a linear positive relationship between the weight-adjusted waist index (WWI) and OA prevalence, but a non-linear relationship between WWI and rheumatoid arthritis (RA) prevalence (24). These contrasting findings highlight the need for further studies to confirm our results and to explore the underlying mechanisms that may explain these relationships.

The associations observed between the VAI and the risk of OA were notable, albeit modest, and remained consistent across the various subtypes of the condition. Additionally, these associations were stable across different demographic categories, including age, race, smoking status, and presence of diabetes. Although the exact mechanisms linking VAI to OA are not fully understood, several plausible explanations offer insights into this relationship.

First, the accumulation of visceral fat increases the mechanical load on the weight-bearing joints, particularly the knees and hips. This additional pressure contributes to the deterioration of articular cartilage by accelerating wear and tear while also stimulating subchondral bone remodeling and hardening (35). Secondly, visceral adipose tissue secretes a range of bioactive molecules, including adipokines such as leptin and lipocalin, and inflammatory mediators such as IL-1β, TNF-α, CRP, and IL-6 (36). These substances trigger synovial inflammation and exacerbate cartilage breakdown by promoting the production of matrix metalloproteinases, which accelerate the degenerative processes associated with OA. Furthermore, obesity activates NLRP3 inflammasomes, leading to the release of proinflammatory cytokines that further worsen OA symptoms (37).

Third, visceral fat plays a crucial role in the development of metabolic disorders such as type 2 diabetes, which can influence OA progression. Elevated blood glucose levels increase oxidative stress and formation of advanced glycation end products (AGEs) in chondrocytes, potentially contributing to joint damage (38). In addition, obesity has been linked to changes in the gut microbiome, leading to intestinal permeability. This allows bacterial lipopolysaccharides to enter the bloodstream and trigger systemic inflammation, which may also affect OA pathogenesis (39).

Obesity is a multifaceted condition that impacts various fat depots, including visceral adiposity, subcutaneous adiposity, bone marrow adiposity, and ectopic fat accumulation in organs such as the liver, pancreas, and skeletal muscle. While our study focused on the VAI as a measure of visceral adiposity and found a significant association between higher VAI and increased OA risk, it is crucial to recognize that other fat depots may also contribute to OA risk. For instance, bone marrow adiposity has been linked to lower bone density and higher fracture risk, and may secrete inflammatory cytokines that contribute to joint inflammation and OA progression (40). Ectopic fat accumulation, such as hepatic steatosis and intramyocellular lipid accumulation, can lead to metabolic dysfunction and inflammation, which are also risk factors for OA (41, 42). Although our study did not directly measure these fat depots, future research should explore their potential roles in OA pathogenesis.

Overall, our findings highlight the need for further research to confirm these associations, deepen our understanding of the complex relationship between VAI and OA, and explore the mechanisms driving this connection.

The strengths of our study stem from the thorough analysis of the NHANES data, incorporating appropriate sample weights, which improved the statistical power to examine the link between VAI and OA. Additionally, we adjusted for relevant covariates to control confounding factors, thereby enhancing the robustness and generalizability of our findings to a wider population. However, this study had several limitations. First, the cross-sectional design of the study limits the ability to infer causality between VAI and OA and does not consider the possibility of reverse causality, where OA may lead to increased VAI associated with reduced physical activity and metabolic changes. This highlights the need for future prospective studies and intervention trials to establish a causal relationship. Therefore, the inherent constraints of observational research must be considered when interpreting results. Second, although we controlled for all known confounders in our multivariate analysis, the possibility of residual or unmeasured confounders remained, which could lead to an overestimation of the observed associations.

## Conclusion

This study indicated that higher VAI values are linked to an increased risk of OA in individuals aged 50 years. Our results introduce new potential predictors that could inform strategies for the prevention and treatment of OA. However, additional large-scale prospective studies are necessary to clarify the mechanisms underlying this association.

## Data Availability

Publicly available datasets were analyzed in this study. This data can be found at: https://www.cdc.gov/nchs/nhanes/index.html.

## Footnotes

1
http://www.R-project.org

2
http://www.clinicalscientists.cn/freestatistics
